# Host microRNAs are decreased in pediatric solid-organ transplant recipients during EBV+ Post-transplant Lymphoproliferative Disorder

**DOI:** 10.3389/fimmu.2022.994552

**Published:** 2022-10-07

**Authors:** Ayantika Sen, Jeanna Enriquez, Mahil Rao, Marla Glass, Yarl Balachandran, Sharjeel Syed, Clare J. Twist, Kenneth Weinberg, Scott D. Boyd, Daniel Bernstein, Amber W. Trickey, Dita Gratzinger, Brent Tan, Mary Gay Lapasaran, Mark A. Robien, Merideth Brown, Brian Armstrong, Dev Desai, George Mazariegos, Clifford Chin, Thomas M. Fishbein, Robert S. Venick, Akin Tekin, Heiner Zimmermann, Ralf U. Trappe, Ioannis Anagnostopoulos, Carlos O. Esquivel, Olivia M. Martinez, Sheri M. Krams

**Affiliations:** ^1^ Department of Surgery, Stanford University School of Medicine, Stanford, CA, United States; ^2^ Department of Pediatric Oncology, Roswell Park Comprehensive Cancer Center, Buffalo, NY, United States; ^3^ Department of Pediatrics, Stanford University School of Medicine, Stanford, CA, United States; ^4^ Department of Pathology, Stanford University School of Medicine, Stanford, CA, United States; ^5^ Division of Allergy Immunity Transplantation, National Institute of Allergy and Infectious Diseases, Rockville, MD, United States; ^6^ Rho Federal Systems Division, Rho, Durham, NC, United States; ^7^ Division of Surgical Transplantation, University of Texas (UT) Southwestern Medical Center, Dallas, TX, United States; ^8^ Department of Pediatrics, University of Pittsburgh Medical Center (UPMC) Children’s Hospital, Pittsburgh, PA, United States; ^9^ Department of Pediatrics and Cincinnati Children’s Hospital, University of Cincinnati, Cincinnati, OH, United States; ^10^ Departments of Surgery and Pediatrics, MedStar Georgetown University Hospital, Washington, DC, United States; ^11^ Department of Pediatric Gastroenterology, David Geffen School of Medicine, University of California Los Angeles, Los Angeles, CA, United States; ^12^ Department of Surgery, University of Miami Miller School of Medicine, Miami, FL, United States; ^13^ Department of Internal Medicine II: Hematology and Oncology, DIAKO Ev. Diakoniekrankenhaus, Bremen, Germany; ^14^ Department of Internal Medicine II: Hematology and Oncology , University Medical Center Schleswig-Holstein, Kiel, Germany; ^15^ Department of Pathology, Charité - Universitätsmedizin Berlin, Berlin, Germany; ^16^ Institute of Pathology, University of Würzburg, Würzburg, Germany

**Keywords:** Post-Transplant Lymphoproliferative Disorder, microRNA, extracellular vesicles, Epstein-Barr Virus, solid-organ transplant

## Abstract

Post-transplant lymphoproliferative disorder (PTLD) is a serious complication of solid organ transplantation. Predisposing factors include primary Epstein-Barr virus (EBV) infection, reactivation of EBV in recipient B cells, and decreased T cell immunity due to immunosuppression. In our previous studies EBV infection was demonstrated to markedly alter the expression of host B cell microRNA (miR). Specifically, miR-194 expression was uniquely suppressed in EBV+ B cell lines from PTLD patients and the 3’untranslated region of IL-10 was determined to be targeted by miR-194. Although EBV has been shown to regulate host miR expression in B cell lymphoma cell lines, the expression of miRs in the circulation of patients with EBV-associated PTLD has not been studied. The objective of this study was to determine if changes in miR expression are associated with EBV+ PTLD. In this study, we have shown that miR-194 is significantly decreased in EBV+PTLD tumors and that additional miRs, including miRs-17, 19 and 106a are also reduced in EBV+PTLD as compared to EBV-PTLD. We quantitated the levels of miRs-17, 19, 106a, 155, and 194 in the plasma and extracellular vesicles (EV; 50-70 nm as determined by nanoparticle tracking analysis) from pediatric recipients of solid organ transplants with EBV+ PTLD+ that were matched 1:2 with EBV+ PTLD- pediatric transplant recipients as part of the NIH-sponsored Clinical Trials in Organ Transplantation in Children, (CTOTC-06) study. Levels of miRs-17, 19, 106a, and 194 were reduced in the plasma and extracellular vesicles (EV) of EBV+ PTLD+ group compared to matched controls, with miRs-17 (p = 0.034; plasma), miRs-19 (p = 0.029; EV) and miR-106a (p = 0.007; plasma and EV) being significantly reduced. Similar levels of miR-155 were detected in the plasma and EV of all pediatric SOT recipients. Importantly, ~90% of the cell-free miR were contained within the EV supporting that EBV+ PTLD tumor miR are detected in the circulation and suggesting that EVs, containing miRs, may have the potential to target and regulate cells of the immune system. Further development of diagnostic, mechanistic and potential therapeutic uses of the miRs in PTLD is warranted.

## Introduction

Post-transplant lymphoproliferative disorder (PTLD) is a major complication of immunosuppressive therapy after solid organ transplantation (SOT) and is associated with significant mortality. PTLD includes a heterogeneous group of lymphocytic proliferations, however, a majority of PTLD cases are of B-cell origin and are associated with Epstein-Barr virus (EBV) infection (1, 2). While there is a clear association between EBV and the development of PTLD, our understanding of how EBV infection leads to PTLD remains incomplete.

EBV is a B-cell lymphotropic γ-herpesvirus that infects more than 90% of the adult population worldwide (3). In healthy children, primary infection may either go undetected or be manifested as a mild upper respiratory tract infection. If primary infection occurs in young adults, the symptoms may be more severe, and result in infectious mononucleosis (IM) (4). During primary infection the virus targets the epithelial cells of the nasopharyngeal region, where it rapidly replicates, lysing the host cells and releasing virions that infect neighboring cells. As the virus reaches the secondary lymphoid tissues, it infects B cells that enter the circulation, releasing more virions which go onto infect other subsets of B cells, including memory B cells. The latent phase is a state of dormancy, where the viral genome exists as a nuclear episome that replicates only once per host cell division with only a distinct subset of viral genes expressed.

The ability of EBV to evade immune surveillance has significant effects in immunocompromised hosts, e.g., SOT recipients. One mechanism that has been identified in immunocompromised hosts is through modulation of host miR expression. miRs are small, non-coding, and highly conserved RNAs that regulate gene expression, cell cycle, apoptosis, immune cell development and differentiation by binding to target mRNA and inhibiting translation of the mRNA (5, 6). LMP1 and LMP2A upregulate host miR-155 in EBV-associated nasopharyngeal carcinoma (NPC) cell lines and clinical samples (7). We have also reported the upregulation of miR-155 and its target FOXO3a by overexpression of LMP1 in B-cell lymphoma cell lines (8). Tumorigenic roles of miRs encoded by cell-cycle regulatory gene cluster, miR-17~92, have been reported. Overexpression of miR-18a, encoded by miR-17~92, causes tumor formation in EBV-associated NPC cell lines and mouse models of NPC (9). Other studies have reported the upregulation of miR-21 (encoded by miR-17~92), by LMP1 in NPC cell lines (10) and in EBV-negative diffuse large B cell lymphoma (DLBCL) cell lines transfected with EBNA2 (11). We previously demonstrated that EBV specifically suppresses miR-194 levels which promotes proliferation of EBV+ B cell lymphoma cell lines *via* increased production of IL-10 (12). Although EBV has been shown to modulate host miRs there is a paucity of studies that quantitate miR expression in the circulation of patients with EBV-associated PTLD. Therefore, the objective of this study was to determine if changes in miR expression are associated with EBV+ PTLD in SOT patients. As part of a prospective multi-institutional study intended to identify viral and immune biomarkers of EBV-associated PTLD, we examined the expression of five miRs, previously shown by our group and others to be altered by EBV, in plasma obtained from 22 pediatric SOT recipients with biopsy-proven EBV+ PTLD and 43 matched-controls. Our results clearly demonstrate that significant alterations in miRs are seen during EBV+PTLD and that these miRs are contained in circulating extracellular vesicles.

## Materials and methods

### Patient samples

#### Blood samples

Blood was obtained from pediatric SOT recipients enrolled at seven sites in the NIAID-sponsored Clinical Trials of Organ Transplantation in Children (CTOTC)-06 (ClinicalTrials.gov Identifier: NCT02182986), a prospective multi-institutional study intended to identify viral and immune biomarkers of EBV-associated PTLD (**Supplement Figure S1**). This study was approved by the Institutional Review Board at each of these sites: Lucile Packard Children’s Hospital Stanford, CA; University of Texas Southwestern Medical Center, Dallas, TX; University of Pittsburgh Medical Center Children’s Hospital, Pittsburgh, PA; University of California, Los Angeles, CA; University of Miami Health System, Miami, FL; Medstar Georgetown Transplant Institute, Washington, DC; Cincinnati Children’s Hospital Medical Center, University of Cincinnati, Cincinnati, OH. All participants provided written informed consent prior to inclusion in the study. Prospective blood samples were collected (n=4753) at enrollment or transplant, every three months during the first two years, and twice yearly thereafter at the time of primary EBV infection, at PTLD diagnosis and during the intense monitoring post-PTLD diagnosis. Participants were followed for a minimum of 12 months and a maximum of four years based on the time of enrollment. Participants enrolled post-transplant (61%) were enrolled within three years of transplantation. EBV serology was performed at the time of transplantation to determine donor and recipient serostatus. EBV PCR to assess viral load was performed by each center’s local laboratory. The primary endpoint was the development of PTLD during the study period with 34 participants reaching the endpoint. Matched controls were selected from the participant pool according to the following criteria: organ type, age, confirmed EBV positive status during sample collection, and post-transplant sample availability proximal to the time of PTLD diagnosis for the PTLD+ subjects. Additionally, for case and control participants who were EBV negative at transplant, controls were identified based on (i) the similarity of post-transplant time-to-first detected EBV DNA (DNAemia) to the corresponding PTLD case and (ii) control sample availability at a similar time post-transplant as the corresponding date of PTLD diagnosis. In this study, based on sample availability and timing, plasma samples obtained from 22 EBV+ pediatric SOT recipients with biopsy-proven PTLD and 43 matched controls (EBV+ pediatric SOT recipients with no PTLD development) were analyzed. Each PTLD+ patient was matched with 1-3 control patients. The ages of participants ranged between 6 months and 19 years with the average age being 7.1 years. About 55% of the patients were females and 45% were males. The types of organ transplant received by participants were heart (n=17), kidney (n=14), liver (n=22), liver and small intestines (n=2), small intestines (n=3) and total visceral transplant (n=7). The WHO classification was used to assign pathological categories, nine of the PTLD+ subjects had early lesions while nine had monomorphic (DLBCL) and four had polymorphic lesions. The average time of PTLD diagnosis post-transplant was 22.5 months (**Table 1**). Researchers conducting this study were blinded to the PTLD diagnosis. Plasma was isolated, aliquoted and frozen until analyzed.

**Table 1:** Clinical information for pediatric Solid Organ Transplant recipient cohort.

Patient #	Age (yrs)	Sex	Solid Organ Transplant	Time from Transplant to PTLD (months)	EBV	PTLD	Histology	Alive/Dead	Cause of Death
1	4	F	Liver	17.9	pos	pos	Monomorphic	alive	NA
2	12	F	Total visceral transplant	1.7	pos	pos	Monomorphic/DLBCL	dead	Multiorgan dysfunction and sepsis
3	19	F	Total visceral transplant	3.9	pos	pos	Monomorphic/DLBCL	alive	NA
4	0.9	F	Heart	33	pos	pos	Early lesion	alive	NA
5	4	F	Kidney	10.8	pos	pos	Polymorphic	alive	NA
6	14.5	M	Heart	12.9	pos	pos	Monomorphic/DLBCL	alive	NA
7	0.6	M	Modified multi visceral transplant (stomach, small bowel and colon)	5.4	pos	pos	Monomorphic/DLBCL	alive	NA
8	7	M	Heart	43.6	pos	pos	Monomorphic/DLBCL	alive	NA
9	2	M	Liver	9.4	pos	pos	Early lesion	alive	NA
10	15	M	Heart	5.3	pos	pos	Polymorphic	dead	Cardiac arrest due to sepsis due to bowel perforation due to DLBCL of GI tract
11	2	F	Liver	37.3	pos	pos	Early lesion	alive	NA
12	16	F	Liver	28.4	pos	pos	Monomorphic/DLBCL	alive	NA
13	16	F	Kidney	28.7	pos	pos	Polymorphic	alive	NA
14	8	F	Kidney	3.1	pos	pos	Polymorphic	alive	NA
15	2	M	Kidney	7.7	pos	pos	Early lesion	alive	NA
16	12	M	Heart	20.1	pos	pos	Early lesion	alive	NA
17	4	M	Liver with small intestines	40.8	pos	pos	Early lesion	alive	NA
18	6	F	Liver	9.6	pos	pos	Early lesion	alive	NA
19	5	F	Liver	50.1	pos	pos	Monomorphic/DLBCL	alive	NA
20	4	F	Liver	76.7	pos	pos	Monomorphic/DLBCL	dead	Relapsed/progressive PTLD, Burkitt’s Lymphoma
21	2	M	Liver	11.6	pos	pos	Early lesion	alive	NA
22	11	F	Heart	27.5	pos	pos	Early lesion	alive	NA
23	2	F	Liver	NA	pos	neg	NA	alive	NA
24	1	M	Liver	NA	pos	neg	NA	alive	NA
25	3	F	Liver	NA	pos	neg	NA	alive	NA
26	18	M	Heart	NA	pos	neg	NA	alive	NA
27	3	F	Total visceral transplant	NA	pos	neg	NA	alive	NA
28	6	M	Kidney	NA	pos	neg	NA	alive	NA
29	3	F	Kidney	NA	pos	neg	NA	alive	NA
30	13	F	Liver	NA	pos	neg	NA	alive	NA
31	10	F	Heart	NA	pos	neg	NA	alive	NA
32	11	F	Heart	NA	pos	neg	NA	alive	NA
33	8	M	Small intestine	NA	pos	neg	NA	alive	NA
34	12	M	Liver with small intestines	NA	pos	neg	NA	dead	Multiorgan failure
35	1	M	Small intestine	NA	pos	neg	NA	alive	NA
36	12	F	Heart	NA	pos	neg	NA	alive	NA
37	12	F	Small intestine	NA	pos	neg	NA	alive	NA
38	5	M	Heart	NA	pos	neg	NA	alive	NA
39	5	M	Modified multi visceral transplant (stomach, small bowel and colon)	NA	pos	neg	NA	alive	NA
40	9	M	Liver	NA	pos	neg	NA	alive	NA
41	8	M	Liver	NA	pos	neg	NA	alive	NA
42	5	F	Kidney	NA	pos	neg	NA	alive	NA
43	8	F	Kidney	NA	pos	neg	NA	alive	NA
44	8	F	Kidney	NA	pos	neg	NA	alive	NA
45	8	M	Liver	NA	pos	neg	NA	alive	NA
46	4	F	Kidney	NA	pos	neg	NA	alive	NA
47	1	F	Heart	NA	pos	neg	NA	alive	NA
48	5	F	Kidney	NA	pos	neg	NA	alive	NA
49	0.6	F	Liver	NA	pos	neg	NA	alive	NA
50	15	M	Heart	NA	pos	neg	NA	alive	NA
51	5	M	Total visceral transplant	NA	pos	neg	NA	alive	NA
52	1	F	Total visceral transplant	NA	pos	neg	NA	alive	NA
53	2	M	Heart	NA	pos	neg	NA	alive	NA
54	16	F	Kidney	NA	pos	neg	NA	alive	NA
55	2	M	Liver	NA	pos	neg	NA	alive	NA
56	16	M	Liver	NA	pos	neg	NA	alive	NA
57	15	F	Liver	NA	pos	neg	NA	alive	NA
58	11	F	Kidney	NA	pos	neg	NA	alive	NA
59	2	F	Heart	NA	pos	neg	NA	alive	NA
60	5	M	Liver	NA	pos	neg	NA	alive	NA
61	3	F	Liver	NA	pos	neg	NA	alive	NA
62	8	M	Liver	NA	pos	neg	NA	alive	NA
63	10	M	Heart	NA	pos	neg	NA	dead	Cerebro-vascular accident
64	0.6	F	Heart	NA	pos	neg	NA	alive	NA
65	10	M	Kidney	NA	pos	neg	NA	alive	NA

NR, Not Reported; NA, Not Applicable; M, Male; F, Female; Pos, positive; Neg, negative; DLBCL, Diffuse Large B-cell Lymphoma.

#### PTLD tumor sections

Tissue sections were obtained from 24 adult SOT cases of PTLD, diagnosed at the Department of Pathology, Charité Universitätsmedizin Berlin, between June 1994 and May 2013. Corresponding clinical data were obtained from the German PTLD registry. The German PTLD registry is a prospective registry that had been initiated in Germany in 2006 to assess the clinical features, treatment options and outcome of PTLD. Data is collected in before, during and at least at 4 weeks, 6, 12 and 24 months after treatment. The ethics committee of the University Medical Center Schleswig-Holstein, Campus Kiel, Germany approved the registry, and all patients gave written informed consent according to the Declaration of Helsinki. Disease stage at enrollment was determined through a full patient history, physical examination, laboratory investigations (including complete blood count, serum lactate dehydrogenase activity, and renal and liver function tests), bone marrow biopsy, and computed tomography scans of the head, chest, and abdomen. The diagnosis of PTLD was based on the examination of formalin-fixed, paraffin-embedded (FFPE) tissue specimens, obtained either by open biopsy or core needle biopsy. All diagnostic tissue samples including conventional histology (i.e., hematoxylin and eosin and Giemsa stains) and immunohistochemistry were reviewed by an expert pathologist and classified according to the criteria of the 2008 WHO classification (13). EBV was confirmed in 14 PTLD cases by *in-situ* hybridization for EBERs (**Supplement Table S1**).

### RNA isolation from plasma and qPCR

The mirVana™ PARIS™ Kit (AM1556, Invitrogen, Waltham, MA) was used to isolate total RNA from plasma obtained from 65 pediatric SOT recipients. The plasma samples were treated with cell dissociation buffer supplied with the mirVana™ PARIS™ Kit to capture miRs encapsulated in EVs of the plasma. The remaining steps performed followed the manufacturer’s protocol. Removal of genomic DNA was carried out using TURBO DNA-free™ Kit, AM1907, Invitrogen, Waltham, MA) following the manufacturer’s protocol. RNA samples were purified and concentrated using RNA Clean & Concentrator-25 (R1017, Zymo Research, Irvine, CA) following the manufacturer’s protocol. RNA concentration and purity were determined using SpectraMax^®^ i3x (Molecular Devices, San Jose, CA).

Concentrations of miRs were determined by qPCR as detailed above. For each sample, 7.0 ng of purified total RNA was used to synthesize miR-specific cDNA using TaqMan™ MicroRNA Reverse Transcription Kit (4366596, Invitrogen, Waltham, MA) and TaqMan^®^ primers (Thermofisher, Waltham, MA) following manufacturer’s protocol.

Expression of miR-17, miR-19, miR-106a, miR-155 and miR-194 in the patient samples were determined by qPCR. The qPCR reaction mix in each well of a 384-well plate consisted of 0.50 µl of 20X qPCR TaqMan^®^ Primers (Catalog no. 4427975, Thermofisher Scientific, Waltham, MA), hsa-miR-17 (Assay ID: 002308), hsa-miR-19 (Assay ID: 000395), hsa-miR-106a (Assay ID: 002169), hsa-miR-155 (Assay ID: 002623), or hsa-miR-194 (Assay ID: 000493); 2X Maxima Probe/ROX qPCR Master Mix (Catalog no. K0231, Thermofisher Scientific, Waltham, MA). A standard curve was generated using Universal miR Reference Kit (Catalog no. 750700, Agilent Technologies, Santa Clara, CA) to quantify each target miR. All experiments were conducted in triplicate. Kruskal-Wallis test and Dunn’s multiple comparison test were performed using GraphPad Prism 9 (GraphPad Software, Inc., La Jolla, CA).

### Isolation of extracellular vesicles from plasma samples

Based on sample availability, EVs were isolated from 250 µl aliquots of plasma collected from 18 EBV+ PTLD+ and 37 EBV+ PTLD- pediatric SOT recipients. Isolation was carried out using ExoQuick Plasma Prep with Thrombin (EXOQ5TM-1 System Biosciences, Palo Alto, CA) following the manufacturer’s protocol. The EV pellets were used for further analysis of miR expression. Supernatants from some samples were retained to analyze miR levels in EV-depleted plasma. The EV pellets and supernatants from some plasma samples were subject to nanoparticle tracking analysis (NTA) to confirm if the isolation of EVs was successful. NTA was performed by System Biosciences, Palo Alto, CA.

### RNA isolation from extracellular vesicles and qPCR

Total RNA was isolated from EV using the SeraMir Exosome RNA Purification Column Kit (Catalog no. RA808A-1, System Biosciences, Palo Alto, CA) following the manufacturer’s protocol. Genomic DNA was removed from isolated RNA samples using TURBO DNA-free™ Kit (AM1907, Invitrogen, Waltham, MA) following the manufacturer’s protocol. RNA samples were purified and concentrated using RNA Clean & Concentrator-25 (R1017, Zymo Research, Irvine, CA) following the manufacturer’s protocol. Concentration and purity were determined using SpectraMax^®^ i3x (Molecular Devices, San Jose, CA). Expression of miR-17, miR-19, miR-106a, miR-155 and miR-194 in the patient samples were determined by qPCR as detailed above. Kruskal-Wallis test and Dunn’s multiple comparison test were performed using GraphPad Prism 9 (GraphPad Software, Inc., La Jolla, CA).

### RNA Isolation, miR microarray, and qPCR from tissue

Total RNA was isolated from PTLD tissue samples obtained from 24 adult cases of PTLD using the RecoverAll™ Total Nucleic Acid Isolation Kit (Ambion, Austin, TX). For each patient sample, three 10 µm sections of the FFPE tissues were processed according to the manufacturer’s instructions. RNA concentration and purity were determined with the Nanodrop^®^ ND-1000 UV-Vis Spectrophotometer (Thermo Fisher Scientific Inc., Wilmington, DE).

Expression of human miRs was analyzed in 150 ng of total RNA from nine patient samples, categorized as EBV+ (n = 4) or EBV- (n = 5) PTLD by hybridization on Affymetrix’s GeneChip miR Array 4.0 (Stanford Functional Genomics Facility, Stanford, CA). The Bioconductor ‘oligo’ package was used to perform array background subtraction, quantile normalization, and summarization by median polish (14). The normalized gene expression dataset was annotated with the ‘pd.mirna.4.0’ annotation library package in R (R Core Team) (14). The expression data was fit to a linear model using the ‘stats’ package in R (R Core Team). Moderated t-statistics and log-odds of differential expression were calculated using the empirical Bayes method. False discovery rate (FDR) tests were performed with the Benjamini-Hochberg procedure for multiple testing correction in R (R Core Team).

Expression levels of five miRs (miR-17, miR-19, miR-106a, miR-155, and miR-194) were quantitated from the remaining 15 PTLD patient samples (10 EBV+ and 5 EBV-) qPCR as detailed above. Mann-Whitney test was performed using GraphPad Prism 9 (GraphPad Software, Inc., La Jolla, CA).

## Results

### miR-17 and miR-106a are significantly reduced in the plasma of EBV+ PTLD+ pediatric transplant recipients

**Figure 1:**
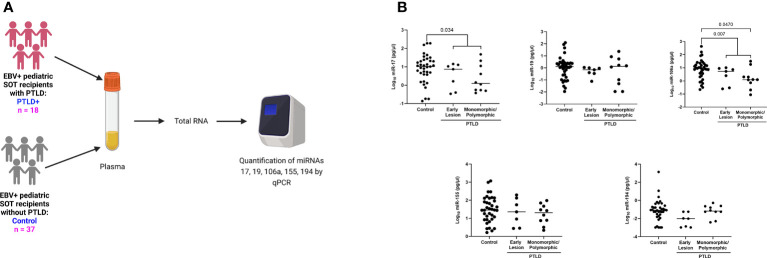
Decreased levels of miRs are detected in the plasma of EBV+ PTLD+ pediatric transplant recipients. **(A)** Schematic diagram of the workflow for quantitation of plasma derived miR from EBV+ PTLD+ pediatric SOT recipients (n = 18) and EBV+ PTLD- matched controls (n = 37) **(B)** Concentrations of miR-17, miR-19, miR-106a, miR-155 and miR-194 were measured by qPCR in plasma of PTLD+ patients with early lesions or monomorphic/polymorphic lesions and matched controls. PTLD+ vs controls, miR-17, p = 0.034; miR-106a, p = 0.007 by Mann-Whitney test. Monomorphic/polymorphic PTLD vs. control, miR-106a, p = 0.047 by Kruskal-Wallis test and Dunn’s multiple comparison test.

It has been clearly demonstrated that infection with EBV has the potential to re-shape the miRNA profile. Indeed, microarray analysis of host miRs from EBV+PTLD and EBV-PTLD tumors, demonstrated that EBV+ PTLD has a distinct miR expression profile as compared to EBV- PTLD (**Supplement Figures S2A, B**). Based on our previous studies we analyzed RNA from an additional 10 EBV+ and 5 EBV- FFPE PTLD tumor blocks for quantitative expression of miRs-17, 19, 106a, 155 and 194. MiRs-17, 19, 106a and 194 trended lower in EBV+ PTLD tumors, with miR-194 being significantly decreased (p = 0.0007) in EBV+ tumors. There were no differences in the expression of miR-155 between EBV+ and EBV- PTLD tumors (**Supplement Figure S2C**). To determine if miRs-17, 19, 106a, 155 and 194 were differentially expressed in the circulation of pediatric transplant recipients based on PTLD status, we analyzed plasma samples from EBV+ pediatric transplant recipients with and without PTLD. PTLD+ cases (n = 18) were matched 1:2 to PTLD- control (n = 37) pediatric transplant recipients by organ transplanted, EBV serostatus, and the time post-transplant (**Figure 1A**). The plasma levels of miRs-17, 19, 106a, and 194 were reduced in pediatric transplant recipients with PTLD as compared to matched controls, with miRs-17 (p = 0.034) and 106a (p = 0.007) being significantly reduced (**Figure 1B**). MiRs-155 expression in plasma did not differ significantly between PTLD+ patients and controls. The type of allograft transplanted did not have a significant impact on miR expression (**Figure S3**). Thus, our data suggest there is regulated expression of miRs in the circulation of children with PTLD.

### Longitudinal analysis of miR expression in plasma indicates a decrease in miR prior to PTLD diagnosis

**Figure 2:**
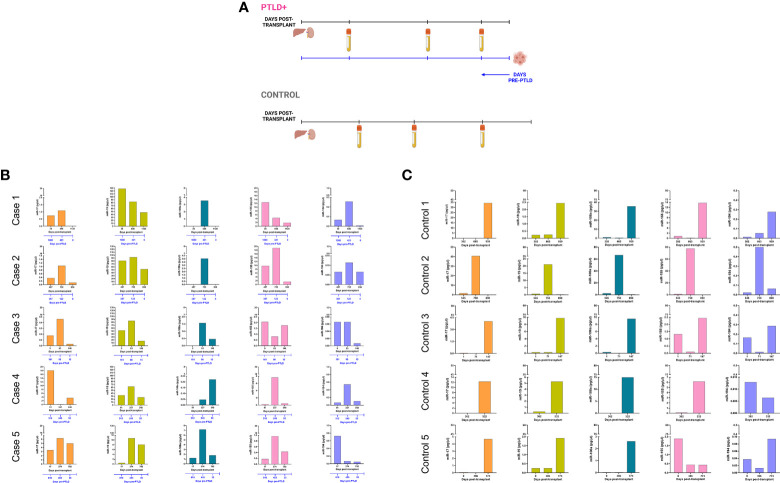
Longitudinal analysis demonstrates a decrease in miR levels prior to a diagnosis of PTLD. **(A)** Schematic diagram of the pairing of PTLD+ (n = 13) and control (n = 13) subjects by matching organ and time of sample collection post-transplant. In both PTLD and control groups, the timeline in black represents the number of days post-transplant. In the PTLD group, the timeline in blue represents the number of days prior to PTLD diagnosis, indicated by Day 0. In the PTLD+ group, the last samples were collected between 0-60 days prior to PTLD diagnosis. **(B)** Concentrations of miR-17, miR-19, miR-106a, miR-155 and miR-194 were measured by qPCR in plasma samples collected at three timepoints post-transplant with the last sample collected within 0-60 days prior to PTLD diagnosis (n = 5). **(C)** Concentrations of miR-17, miR-19, miR-106a, miR-155 and miR-194 were measured by qPCR in plasma samples collected post-transplant from control patients (n = 5) at timepoints matched with paired PTLD+ subjects.

Longitudinal analysis for miRs-17, 19, 106a, 155 and 194 was performed to determine miRNA expression changes between transplantation and PTLD diagnosis. From the cohort described in **Figure 1A**, a subset of EBV+ PTLD+ patients (n=13) were matched 1:1 to EBV+ controls (n=13) by allograft type and number of days post-transplant (**Figure 2A**). Five PTLD+ and five control subjects having at least three timepoints with the last timepoint being within 60 days prior to PTLD diagnosis (for the PTLD+ group) are shown in **Figures 2B, 2C**. We observed a decline in plasma levels of miRs-17, miR-19 and miR-194 in all five EBV+ PTLD+ patients at the timepoint closest to PTLD diagnosis. The plasma levels of miR-106a and miR-155 also declined in four out of five PTLD+ patients.

The remaining eight PTLD+ and eight control patients (**Figure S4**) either had samples collected at only two timepoints or had the last sample collected more than 60 days prior to the day of PTLD diagnosis. The data in these eight pairs also demonstrated a consistent decrease in plasma miR-17 and miR-19 levels over time; furthermore, the samples obtained within 60 days of PTLD diagnosis had the least miR-17 and miR-19 levels. These data suggest that miRs 17, 19, 106a and 194 are decreased prior to a clinical diagnosis of PTLD in EBV+ pediatric transplant recipients.

### miRs-17, 19, 106a, 155 and 194 are localized in circulating extracellular vesicles of EBV+ solid organ transplant recipients

**Figure 3:**
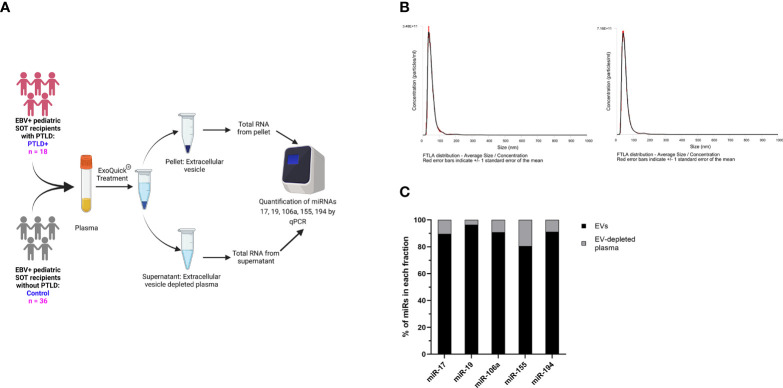
miRs are localized to EVs in plasma. **(A)** Schematic diagram of the workflow for the isolation of EVs from plasma and qPCR analysis of EV-derived miRNA from PTLD (n = 18) and control (n = 36) pediatric SOT recipients **(B)** Characterization of vesicles size and concentration of EVs in EV pellets isolated from plasma fraction by nanoparticle tracking analysis. **(C)** Concentrations of miR-17, miR-19, miR-106a, miR-155 and miR-194 were measured by qPCR in EVs and EV-depleted plasma samples. Black bars represent the fraction of EV-derived miRNAs in whole plasma. Gray bars represent the fraction of miRNAs derived from EV-depleted plasma.

miRs produced by cells can be released into the circulation as either free miRs or encapsulated into EVs, which are then released into the circulation. We quantitated the levels of miRs-17, 19, 106a, 155 and 194 in plasma EVs and EV-depleted plasma from the same cohort of PTLD+ (n=18) and control patients (n=36) described in **Figure 1** (**Figure 3A**). The mean size of EVs in our samples, as determined by NTA, was 60.1 +/- 14.86 nm, consistent with the size distribution of intact exosomes (**Figure 3B**). EV-depleted plasma demonstrated a thousand-fold decrease in the concentration of detected particles as compared to the concentration of detected particles in isolated EV pellets.

More than 90% of total plasma miR-17,-19,-106a, and -194 and more than 80% of total plasma miR-155 were contained within the EV fraction (**Figure 3C**), thus indicating that the majority of these cell-free miRs are sorted into circulating EVs.

### miRs-17, 19 and 106a are significantly reduced in the extracellular vesicles of EBV+ PTLD+ pediatric transplant recipients with monomorphic/polymorphic lesions

**Figure 4:**
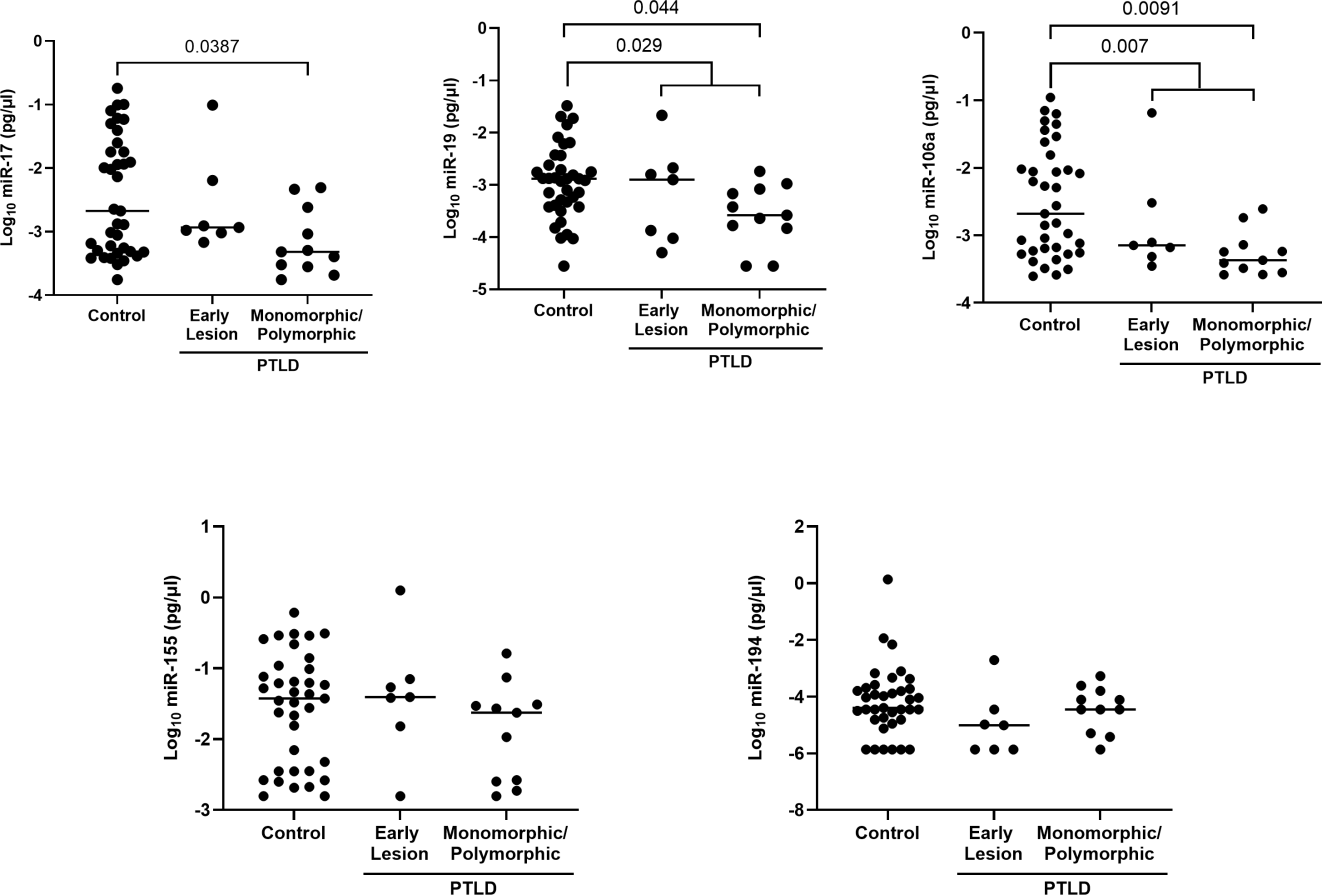
Decreased levels of miRs are detected in the extracellular vesicles of PTLD+ pediatric transplant recipients as compared to controls. Concentrations of miR-17, miR-19, miR-106a, miR-155 and miR-194 were measured by qPCR in isolated EVs from PTLD+ (n = 18) and control (n = 36) patients. PTLD+ vs controls, miR-19, p = 0.029; miR-106a, p = 0.007 by Mann-Whitney test. Monomorphic/polymorphic PTLD vs. control, miR-17, p = 0.0387; miR-19, p = 0.044; miR-106a, p = 0.0091 by Kruskal-Wallis test and Dunn’s multiple comparison test.

The expression of the EV-derived miRs between PTLD+ and control groups revealed a significant reduction in miRs-19 (p = 0.029) and -106a (p = 0.007) levels in children with PTLD+ and a trend towards lower levels of miRs-17 and -194 (**Figure 4**). Further, subjects with monomorphic/polymorphic lesions had even lower levels of miRs 17,19, and 106a than children with early lesions. We observed significant decreases in expression of miRs-17 (p = 0.039), -19 (p = 0.044), and -106 (p = 0.009) in the EVs of patients with monomorphic/polymorphic lesions as compared to controls. Expression of miRs-155 and -194 remained unchanged between PTLD and control groups. Taken together, our results indicate that miRs 17,19 and 106a are decreased during the development of PTLD in EBV+ pediatric SOT recipients.

## Discussion

We have demonstrated that miR-17, miR-19 and miR-106a are decreased in circulating EVs of EBV+ pediatric SOT recipients with monomorphic/polymorphic PTLD. The data on miR expression in PTLD are very limited. A comparative analysis of miR expression between central nervous system-associated PTLD tissues and systemic DLBCL-like PTLD tissues in adult SOT transplant recipients identified a number of miRs, including miRs-17, 19, and 155, that were differentially expressed between the two cohorts (15). To date, however, there has not been an analysis of miR expression in the circulation of SOT recipients with PTLD.

Some forms of PTLD can share histologic characteristics with non-transplant EBV-associated diffuse large B cell lymphoma (DLBCL) or Burkitt’s lymphoma, and there is a growing body of literature on the role of miRs in these histologically-similar lymphomas. miRs-17, 19a, and 106a are encoded by the miR-17~92 family of gene clusters which includes the miR-17~92 cluster and its paralog clusters, miR-106a~363 and miR-106b~25 (16). Expression analysis of human lymphoma samples (both DLBCL and non-DLBCL) revealed upregulation of the miR-17~92 cluster in the lymphoma tissues (17). In another study, 25% of tumor tissues isolated from patients with DLBCL had upregulation of miR-19b compared to patients with reactive lymphoid hyperplasia, while among patients with various types of non-Hodgkin’s lymphoma, higher expression of miR-19b was associated with a reduction in both overall survival and event-free survival (18).

In our study, however, we observed lower levels of miRs-17 and 19 in patients with PTLD compared to controls. This may be because our patients were receiving immunosuppression therapy and may therefore have had higher EBV activity. Overexpression of EBV in the AGS gastric carcinoma cell line is associated with reduced expression of the miR-17~92 cluster *via* activity of the EBV miR BART1-3p and host transcription factor E2F3 (19). We, too, observed that among patients with PTLD, expression of miRs-17, 19, and -106 were all lower in EBV+ patients compared to EBV- patients.

The sequence for miR-106a is carried by the miR-106a~363 cluster (20), a paralog to the miR-17~92 cluster, that has been shown to be regulated by common transcription factors and have overlapping functions with miR-17~92 cluster (16, 21, 22). There are limited studies on the role of miR-106a in PTLD development. However, in a comparative analysis of miR expression between DLBCL tissue, follicular lymphoma (FL) tissue, and healthy lymph nodes, miR-106a expression was significantly higher in DLBCL compared to normal tissue (23). The same study also demonstrated that miR-106a was a top driver of differences in expression patterns between healthy lymph tissues and lymphoma tissues. We, however, observed the opposite effect in our samples; miR-106a expression was lower in PTLD+ patients compared to controls. One possible explanation for this is genetic heterogeneity among histologically-similar tumors. Microarray analysis of miR content from DLBCL tumor samples divides the histologically-similar DLBCL samples into three genetically-distinct groups with differing amounts of miR-106a expression. Samples with lower miR-106a expression also had lower levels of miR-17 and 19, suggesting that the PTLD tissues used in our study may have more similarities to this subset of DLBCL (24).

DLBCL can be divided into two types based on gene expression profiles, germinal center-like (GC) or activated memory-B cell-like (non-GC), with the GC group having a significantly better prognosis compared to non-GC. miR-155 expression levels were lower in the GC group compared to the non-GC group (25). Among children and young adults with Burkitt’s lymphoma, however, the data on miR-155 expression is conflicting. While one study reported high levels of miR-155 in primary Burkitt’s lymphoma tissue (26), others reported that majority of primary Burkitt’s lymphoma tissue did not express miR-155 at detectable levels (27). We did not observe any differences in miR-155 expression in tumors or in the plasma of EBV+ PTLD patients. This may also be the result of immunosuppression therapies, that have been reported to suppress miR-155 expression (28).

The role of miR-194 in EBV-associated cancers and EBV infection is poorly understood. Previously our group reported the EBV regulates the expression of miR-194 in B-cell lymphoma cell lines derived from patients with PTLD (12). In this *in vitro* study, miR-194 was found to be downregulated in five out of six PTLD patient-derived EBV transformed B-cell lymphoma cell lines. Analysis by TargetScan and miRanda showed that miR-194, miR-17 and miR-106a have target sites in the 3’UTR region of *il-10* gene and we found that overexpression of miR-194 reduced IL-10 production. Our previous study showed that IL-10 serves as an autocrine growth factor in EBV+ lymphoblastoid cell lines derived from patients with EBV+ PTLD (29). We have also shown that inducing LMP1 signaling in EBV-negative B-cells increased the production of IL-10 in these cell lines (30). Another study also reported a significantly increased serum IL-10 levels in EBV positive patients at the time of PTLD diagnosis (31). Taken together, these studies suggest that EBV can suppress miR-194 expression, leading to increased production of IL-10 and survival of B-cell lymphoma cell lines. Our findings also show reduced expression of miR-194 in PTLD tissues of EBV+ PTLD patients compared to EBV- PTLD patients.

One of the mechanisms by which miRs can travel through the circulation to distant sites is through encapsulation in EVs. EVs are cell-derived lipid-bilayer nanostructures that carry proteins, lipids and various RNA subtypes from infected cells to remote locations (32) where they fuse with target cells to transfer their miR cargo. These transferred miRs alter gene expression patterns in the target cells, leading to aberrant growth regulation. EBV is known to exploit EVs to serve as carriers for EBV proteins and miRs. When EVs from EBV+ LCLs were labeled with a fluorescent dye and co-cultured with primary immature monocyte-derived dendritic cells, there was an increase in levels of EBV miRs in the dendritic cells; furthermore, HeLa cells co-cultured with EVs from EBV+ LCLs demonstrated repression of EBV target genes (33–37). Our data demonstrate that host miRs can similarly be packaged into EVs and suggest another mechanism by which EBV may regulate gene expression in uninfected cells. These changes in gene expression may have functional consequences for tumor growth as EVs carrying LMP1 from EBV infected DG75 Burkitt’s lymphoma cell lines induce cell proliferation in healthy B cells (38).

In summary, we demonstrate that selected miRs with potential to regulate growth patterns are differentially expressed in plasma of PTLD+ patients. We also demonstrate that these miRs are found in EVs and can travel to distant sites. Lastly, we demonstrate that decreases in expression of miRs-17, 19 and 106a are temporarily associated with the development of PTLD. Future studies should focus on a thorough characterization of the contents of the EVs in patients with PTLD to determine which host miRs are critical for PTLD development, as miRs may serve as biomarkers for development of PTLD as well as targets for novel therapies.

## Data availability statement

The microarray data presented in the supplement material section of this study are deposited in the ArrayExpress repository, accession number E-MTAB-12201.

## Ethics statement

The studies involving human participants were reviewed and approved by the Institutional Review Board at each of these sites: Lucile Packard Children’s Hospital Stanford, CA; University of Texas Southwestern Medical Center, Dallas, TX; University of Pittsburgh Medical Center Children’s Hospital, Pittsburgh, PA; University of California, Los Angeles, CA; University of Miami Health System, Miami, FL; Medstar Georgetown Transplant Institute, Washington, DC; Cincinnati Children’s Hospital Medical Center, University of Cincinnati, Cincinnati, OH. Written informed consent to participate in this study was provided by the participants’ legal guardian/next of kin.

## Author contributions

Data acquisition and analysis: AS, JE, MR, MG, YB, SS, SK. Manuscript preparation: AS, JE, MR, OM, CE, SK. Pathologist: CT, DG. Statistician: AWT, BA. Clinical Research coordinator: ML. Data analysis: KW, SB, DB. Manuscript review: BT, MAR, MB, BA, Sample acquisition: DD, GM, CC, TF, RV, AT, HZ, RT, IA,

## Funding

This study was funded by NIH A1UO1AI104342, UM2AI117870 (Rho), Stanford Maternal and Child Health Research Institute fellowship (M.R.), the Stanford Transplant and Tissue Engineering Center of Excellence fellowship (A.S., M.R.) the Stanford University Jackson Vaughan Critical Care Research Fund (M.R.) and NIH T32 AI007290 (M.G).

## Conflict of interest

Author BA was employed by Rho.

The remaining authors declare that the research was conducted in the absence of any commercial or financial relationships that could be construed as a potential conflict of interest.

## Publisher’s note

All claims expressed in this article are solely those of the authors and do not necessarily represent those of their affiliated organizations, or those of the publisher, the editors and the reviewers. Any product that may be evaluated in this article, or claim that may be made by its manufacturer, is not guaranteed or endorsed by the publisher.
