# Supplementary material for: Host microRNAs are decreased in pediatric solid-organ transplant recipients during EBV+ Post-transplant Lymphoproliferative Disorder
Source: Front Immunol. 2022 Oct 7;13:994552. doi: 10.3389/fimmu.2022.994552 (PMC9595046; doi:10.3389/fimmu.2022.994552)
Supplement: Supplementary file 1 [file DataSheet_1.docx]

Supplementary Material

**Table S1: Clinical information for adult Solid Organ Transplant recipient cohort**

| **Patient #** | **Age (yrs)** | **Sex** | **Solid Organ Transplant** | **Time from Transplant to PTLD (months)** | **CD20** | **EBV** | **Histology** | **AA stage** | **Alive/ Deceased** | **Cause of Death** | **Analyzed by** |
| --- | --- | --- | --- | --- | --- | --- | --- | --- | --- | --- | --- |
| 1 | 17.6 | M | liver | 5.6 | pos | pos | DLBCL | 1 | alive | NA | Microarray |
| 2 | 32.7 | F | kidney | 334.4 | pos | pos | DLBCL | 3 | dead | PTLD | Microarray |
| 3 | 53.4 | F | heart | 8.8 | pos | pos | DLBCL | 4 | dead | PTLD | Microarray |
| 4 | 47 | F | kidney | 5.8 | pos | pos | DLBCL | 2 | alive | NA | Microarray |
| 6 | 63 | M | heart | 106 | pos | pos | DLBCL | 4 | dead | pneumonia | qPCR |
| 7 | 74 | M | heart | 137.5 | pos | pos | Burkitt-PTLD | NR | NR | NA | qPCR |
| 8 | 23.6 | M | kidney | 58.7 | pos | pos | Burkitt-PTLD | 4 | alive | NA | qPCR |
| 9 | 35.7 | M | heart | 2.8 | pos | pos | DLBCL | 4 | dead | Not reported | qPCR |
| 10 | 33.1 | M | heart/lung | 18.1 | pos | pos | DLBCL | 4 | alive | NA | qPCR |
| 11 | 51.3 | F | heart | NR | pos | pos | pCNS-DLBCL | NR | NR | NA | qPCR |
| 12 | 42 | M | heart | 77.1 | pos | pos | MZL | 2 | alive | NA | qPCR |
| 13 | 58 | M | lung | 51 | pos | pos | DLBCL | NR | NR | NA | qPCR |
| 14 | 76.4 | F | kidney | 162.4 | pos | pos | DLBCL | 1 | dead | perforated diverticulitis | qPCR |
| 15 | 57.1 | F | kidney | 162.8 | pos | pos | pCNS-DLBCL | 4 | dead | PTLD | qPCR |
|  |  |  |  |  |  |  |  |  |  | NA |  |
| 17 | 38.7 | M | kidney | 54.3 | pos | neg | DLBCL | 4 | dead | Not reported | Microarray |
| 18 | 36.1 | M | liver | 22.1 | pos | neg | DLBCL | 3 | dead | transplant failure (Hepatitis C) | Microarray |
| 19 | 50.2 | M | kidney | 138.7 | pos | neg | DLBCL | 4 | alive | NA | Microarray |
| 20 | 65.3 | M | liver | 161.6 | pos | neg | DLBCL | 1 | alive | NA | Microarray |
| 21 | 43.1 | F | liver | 5.5 | pos | neg | DLBCL | 2 | dead | PTLD | Microarray |
| 16 | 51.6 | F | kidney | 156.1 | pos | neg | MZL | 1 | alive | NA | qPCR |
| 22 | 65.8 | M | kidney | 108.8 | pos | neg | DLBCL | 1 | alive | NA | qPCR |
| 23 | 65.5 | M | kidney | 9.1 | pos | neg | DLBCL | 1 | alive | NA | qPCR |
| 24 | 46.2 | M | kidney | 55.9 | pos | neg | DLBCL | 1 | dead | organ failure | qPCR |
| 25 | 65 | M | liver | NR | pos | neg | DLBCL | NR | NR | NA | qPCR |

NR – Not Reported

NA – Not Applicable

M – Male

F – Female

Pos – positive

Neg – negative

DLBCL – diffuse large B cell lymphoma

MZL – marginal zone lymphoma

**Figure S1:**


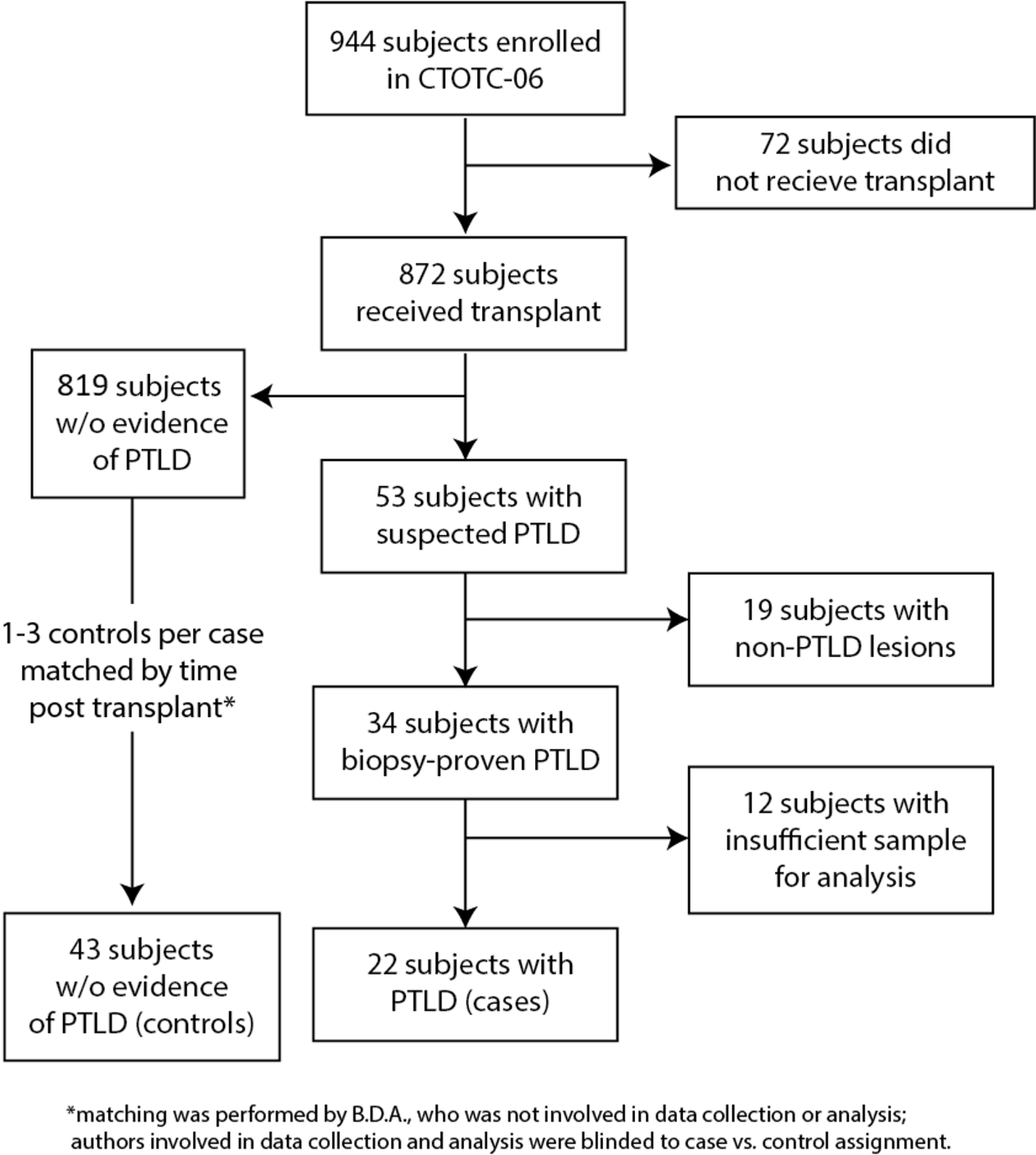


**Figure S1: Inclusion and exclusion strategy for recruitment of subjects in PTLD+ and control groups.** A total of 944 subjects, eligible for receiving transplant, were enrolled for the prospective multi-institutional, NIAID-sponsored Clinical Trials of Organ Transplantation in Children (CTOTC)-06 study. Among the enrolled subjects, 872 subjects received transplant. Post-transplant, 54 subjects were suspected to have developed PTLD but only 34 were declared to have biopsy-proven PTLD. Based on sample availability, 22 subjects with PTLD were included in our study. Among the 819 subjects who did not show any evidence of PTLD, 43 subjects we included in the control group by matching 1-3 control subjects per PTLD+ subject. Matching criteria included organ type, age, confirmed EBV positive status during sample collection, and post-transplant sample availability proximal to the time of PTLD diagnosis for the PTLD+ group.

**Figure S2:**


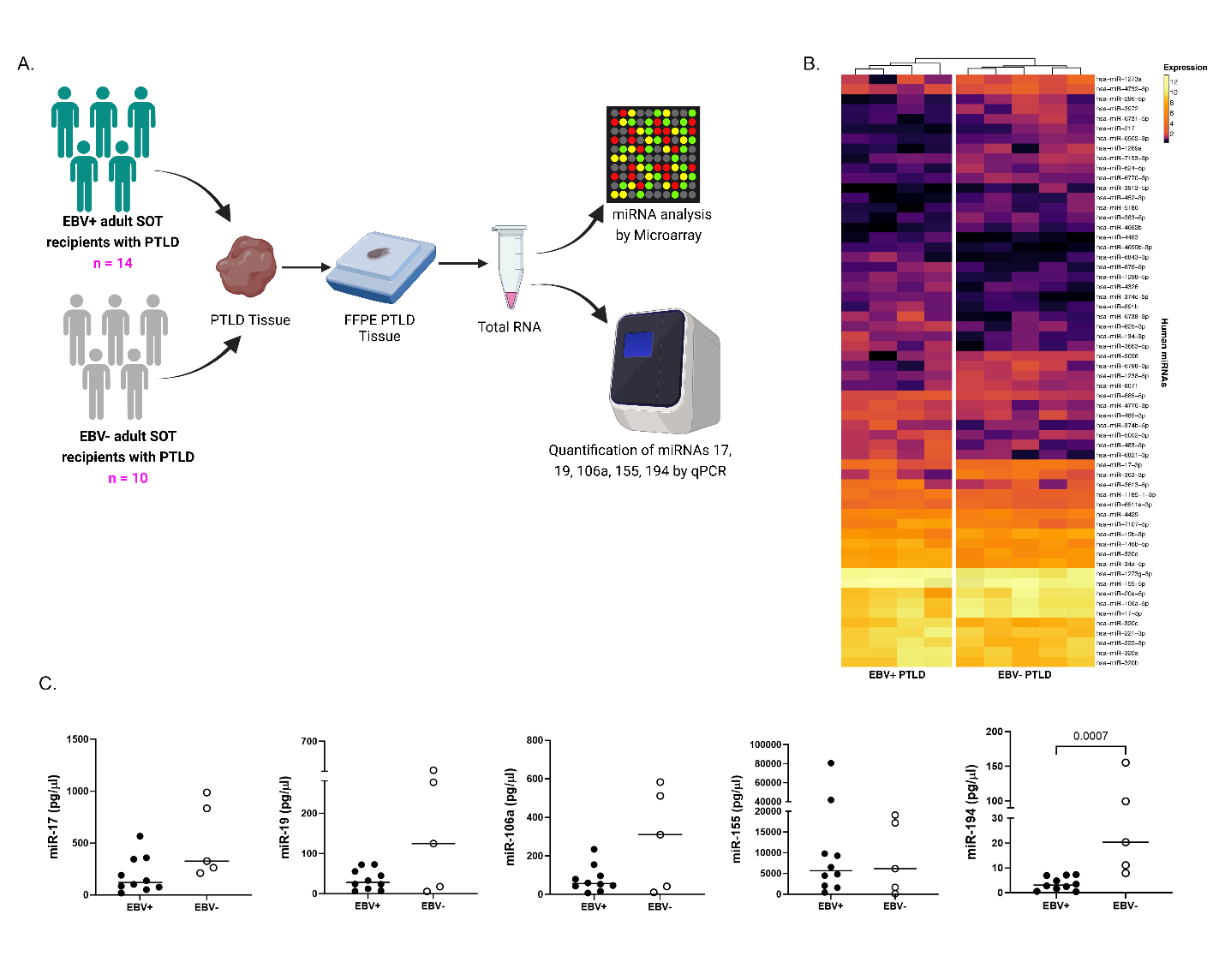


**Figure S2: Human miRs are differentially expressed in EBV+ and EBV- PTLD tumors**. **(A)** Schematic diagram of the workflow for miR analysis of FFPE PTLD tumors from EBV+ and EBV- solid organ transplant recipient. **(B)** Heatmap showing normalized expression values for a subset of human miRs from EBV+ (n = 4) and EBV- (n = 5) PTLD lesions. **(C)** Concentrations of miR-17, miR-19, miR-106a, miR-155 and miR-194 in PTLD tissues were measured by qPCR in a separate cohort of EBV+ (n = 10) and EBV- (n = 5) patients. miR-194, EBV+ vs. EBV-, p = 0.0007 by Mann-Whitney test.

**Figure S3:**


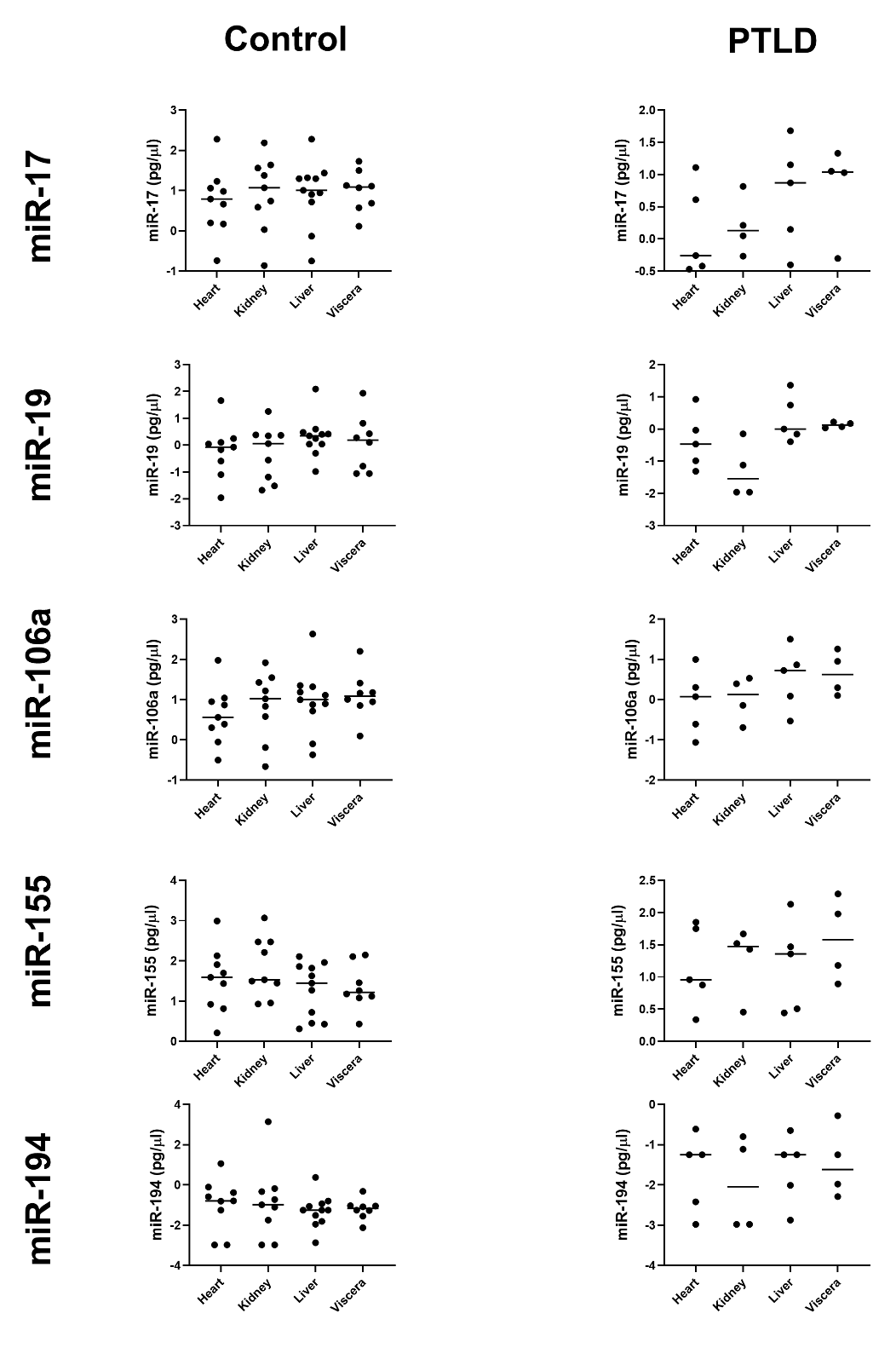


**Figure S3: The plasma miR expression in PTLD group or control group was not impacted by the type of allograft transplanted.** Concentrations of miR-17, miR-19, miR-106a, miR-155 and miR-194 were measured by qPCR in plasma of PTLD+ and PTLD- recipients of heart, kidney, liver and visceral transplant.

**Figure S4:**

**
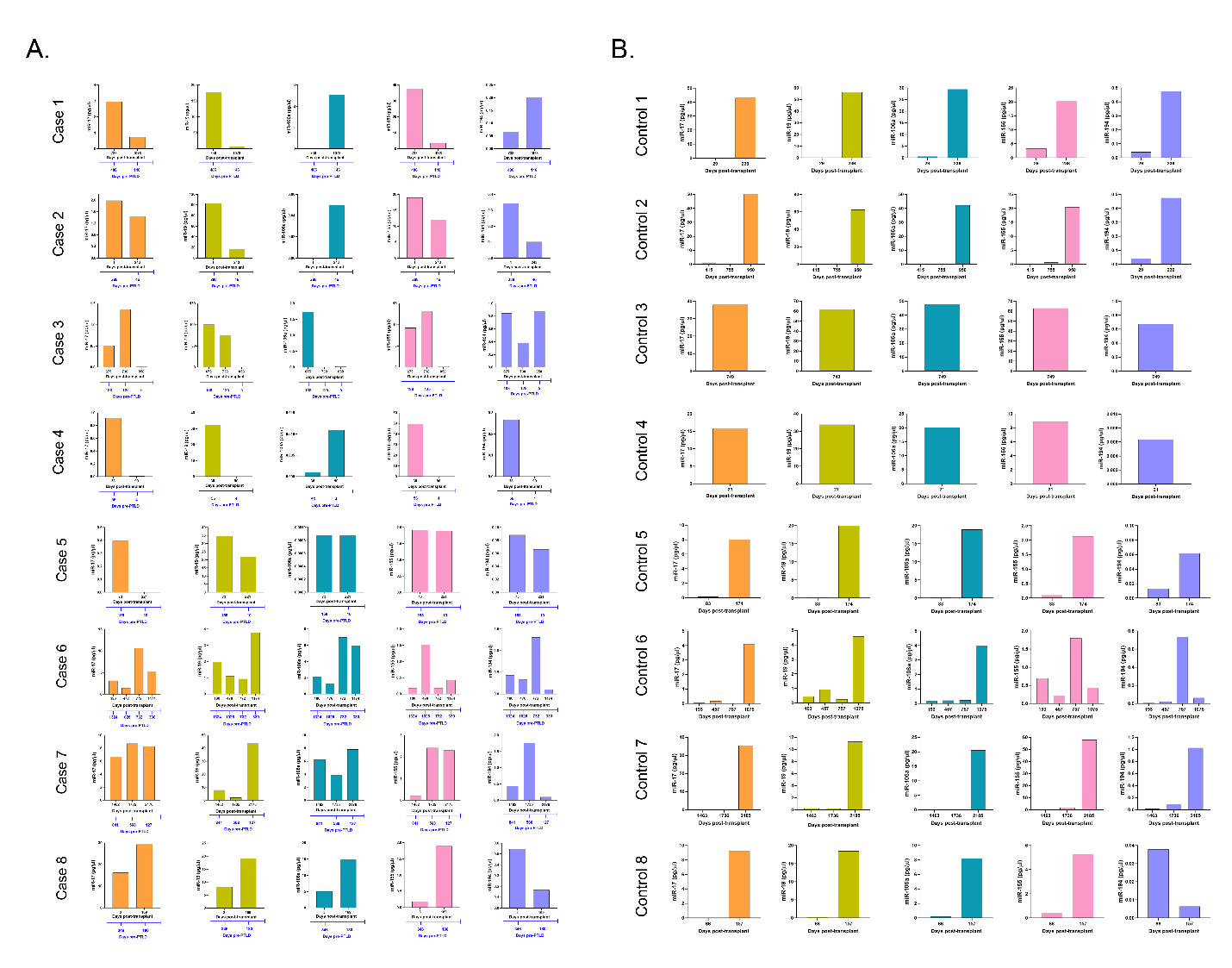
**

**Figure S4: Longitudinal analysis of plasma miRNA expression. (A)** Concentrations of miR-17, miR-19, miR-106a, miR-155 and miR-194 were measured by qPCR in plasma samples collected at multiple timepoints post-transplant from PTLD+ patients (n = 8). **(B)** Concentrations of miR-17, miR-19, miR-106a, miR-155 and miR-194 were measured by qPCR in plasma samples collected post-transplant from control patients at timepoints matched with paired PTLD+ patients (n = 8).
